# Reasons for accepting or declining Down syndrome screening in Dutch prospective mothers within the context of national policy and healthcare system characteristics: a qualitative study

**DOI:** 10.1186/s12884-016-0910-3

**Published:** 2016-05-26

**Authors:** Neeltje M. T. H. Crombag, Hennie Boeije, Rita Iedema-Kuiper, Peter C. J. I. Schielen, Gerard H. A. Visser, Jozien M. Bensing

**Affiliations:** Department of Obstetrics, University Medical Center Utrecht, P.O. Box 85090, Room KE04.123.1, 3508AB Utrecht, The Netherlands; The Netherlands Institute for Health Services Research, Utrecht, The Netherlands; Centre for Infectious Diseases Research, Diagnostics and Screening (IDS), National Institute for Public Health and the Environment (RIVM), Bilthoven, The Netherlands; Faculty of Social and Behavioural Sciences, Utrecht University, Utrecht, The Netherlands

**Keywords:** Down syndrome, Trisomy 21, Prenatal counselling, First trimester combined testing, Down syndrome screening, Prenatal anomaly screening, Informed decision making, Healthcare system characteristics, Focus group study, Qualitative study

## Abstract

**Background:**

Uptake rates for Down syndrome screening in the Netherlands are low compared to other European countries. To investigate the low uptake, we explored women’s reasons for participation and possible influences of national healthcare system characteristics. Dutch prenatal care is characterised by an approach aimed at a low degree of medicalisation, with pregnant women initially considered to be at low risk. Prenatal screening for Down syndrome is offered to all women, with a ‘right not to know’ for women who do not want to be informed on this screening. At the time this study was performed, the test was not reimbursed for women aged 35 and younger.

**Methods:**

We conducted a qualitative study to explore reasons for participation and possible influences of healthcare system characteristics. Data were collected via ten semi-structured focus groups with women declining or accepting the offer of Down syndrome screening (*n* = 46). All focus groups were audio- and videotaped, transcribed verbatim, coded and content analysed.

**Results:**

Women declining Down syndrome screening did not consider Down syndrome a condition severe enough to justify termination of pregnancy. Young women declining felt supported in their decision by perceived confirmation of their obstetric caregiver and reassured by system characteristics (costs and age restriction). Women accepting Down syndrome screening mainly wanted to be reassured or be prepared to care for a child with Down syndrome. By weighing up the pros and cons of testing, obstetric caregivers supported young women who accepted in the decision-making process. This was helpful, although some felt the need to defend their decision to accept the test offer due to their young age. For some young women accepting testing, costs were considered a disincentive to participate.

**Conclusions:**

Presentation of prenatal screening affects how the offer is attended to, perceived and utilised. By offering screening with age restriction and additional costs, declining is considered the preferred choice, which might account for low Dutch uptake rates. Autonomous and informed decision-making in Down syndrome screening should be based on the personal interest in knowing the individual risk of having a child with Down syndrome and system characteristics should not influence participation.

## Background

In the Netherlands, obstetric care is characterised by an approach aimed at a low degree of medicalisation. Pregnant women are initially considered to be at low risk, and most women start their pregnancy with a primary care midwife. As a consequence, most women receive information on Down syndrome screening (DSS) from their midwife, which is called prenatal counselling. Pregnant women receive prenatal counselling during their first visit (around 8 weeks of gestation), after which they make their decision about participation in DSS. The offer to opt for DSS is presented in a way that helps prospective mothers make a well-informed and autonomous choice. In addition, specific training to provide correct information for DSS to pregnant women is required for obstetric caregivers prior to information provision [1]. During the study period (2012–2013), for women aged 36 and over, the test was free of charge, whereas for younger women an additional fee was requested.

First trimester Down syndrome screening is performed by maternal serum screening and by ultrasound measurement of the foetal nuchal translucency in the first trimester of pregnancy, between 9 and 14 weeks of gestation [1]. Down syndrome is the most common chromosomal aneuploidy (trisomy of chromosome 21) with an incidence of about 1 in 700 live births. Down syndrome is associated with intellectual disability, delayed development and certain physical characteristics. Since 2007 DSS in the Netherlands is offered to all pregnant women. The offer is presented with a possibility of ‘the right not to know’ for women who do not want to be informed about DSS [1]. The aim of DSS is to inform prospective parents on the risk of Down syndrome (DS), to provide them with timely options, including invasive diagnostic procedures in the case of increased risk for DS, and if diagnosed, preparation to care for a disabled child or termination of pregnancy (TOP) [1]. In the Netherlands, the overall uptake^1^ for DSS is low (<30 %) when compared to other north-western European countries (74 % in England, 84 % in France and ≥90 % in Denmark) [2–4]. In previous studies, it was suggested that uptake rates are related to the approach adopted by the system in which it is executed. In the Netherlands for example, van den Berg et al. suggested that the low uptake was related to the non-medicalised approach of pregnancy [5]. In contrast, in France high uptake of screening was associated with the tradition of a medicalised approach of pregnancy [6]. The way screening is offered to pregnant women might be impacted by public policies and the health systems in which they function. As suggested in one of our earlier studies and by Bakker et al., the unique characteristics of the Dutch screening programme, such as a financial threshold, and ‘the right not to be informed’ for younger women, might well account for the low uptake rates [7, 8]. To ensure uniform information, it is mandatory for Dutch obstetric caregivers to follow a specialised training on DSS counselling. The ‘right not to know’ is based on the ethical principle of autonomy, giving patients the right to refuse medical information and women are explicitly asked if they want information on DSS, before information is provided. Routinely offering DSS explicitly is not the aim of the Dutch screening programme [8]. Based on observations of information provision, Vassy et al. [6] concluded that these characteristics contributed to equal accessibility to the test without any encouragement to accept it. Therefore there are some indications that healthcare systems factors seem to influence the frequency of utilisation. For example, utilisation of prenatal diagnosis is sensitive to monetary incentives [9] and providing screening during a routine visit results in higher uptake rates as compared to screening offered during a separate visit [10].

While the influence of psychosocial, socio-cultural and individual factors on utilisation of DSS [5, 7, 11–13] (such as beliefs and attitudes towards disability and TOP, religious denomination and ethnic background) have been studied extensively, little is known about the influence of national policies (healthcare system factors) on individual decision-making. To gain a better understanding of the utilisation of DSS, the influence of the external environment (healthcare system and public policy) on individual decision making should be acknowledged [14]. Therefore it is the aim of this study to determine whether the specific Dutch screening policy and healthcare system influence individual decision-making and to investigate if these factors may explain the low DSS uptake rates in the Netherlands. We therefore explored which factors impeded or facilitated the use of DSS, and if and how system characteristics played a role in these considerations.

## Methods

**Fig. 1:**
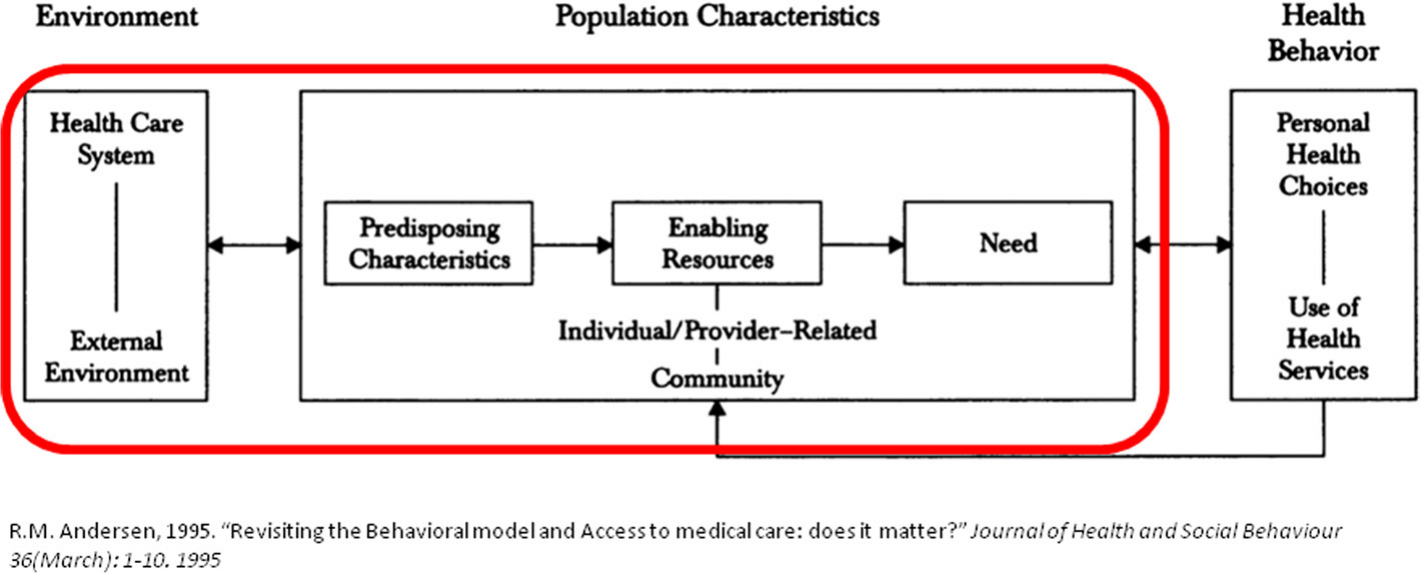
The Behavioral Model of Health Services Use

One of the most frequently used frameworks for analysing utilisation of healthcare services is the behavioural model designed by Andersen (The Behavioral Model of Health Services Use, Fig. 1) [14]. We used the model to understand utilisation of DSS and its interaction with healthcare system characteristics. The model consists of three groups of factors: predisposing factors (such as health beliefs), need factors (objective and/or perceived health/risk) and enabling factors (e.g. availability and accessibility), thus covering factors at both individual and health system level, as well as their mutual interaction. Moreover, it evaluates the influence of all these factors on subsequent healthcare.

To address the research questions, a focus group approach and qualitative analysis were used.

### Recruitment and sample characteristics

Pregnant women were recruited from four primary care practices and from the primary care practice residing in the University Medical Center of Utrecht. Because we anticipated on differences between women that had already received oral information on DSS and women who had not, we used separate recruitment strategies. Women were approached by their community midwife either before their first visit (defined as pre-counselling) or after their first visit (defined as post-counselling). Inclusion criteria for participation included maternal age 18 or over, good understanding of the Dutch language, and singleton pregnancy (if known). Women with a history of foetal anomalies, a current pregnancy with foetal anomalies, maternal complications (a pregnancy identified at risk, according to national guidelines (List of Obstetric Complications, the so-called Verloskundige Indicatie Lijst)) or whose pregnancy was conceived by assisted reproductive technology were excluded. To prevent test experience bias, only participants who had not yet received results of DSS or a foetal anomaly scan were included.

Due to a relatively small period in which we were able to include pregnant women and individual availability, recruitment was a challenge. Over 350 women were approached, of whom 46 participated in the focus groups. The main reasons for non-participation were unsuitable date/time, miscarriage, language barrier, or because they already had had the test results or a foetal anomaly scan at the date of the session.

Women were given information orally, and those interested gave consent to be contacted by the researcher with details about the study. Study personnel recruited participants by phone and gave detailed information about the study. Women who expressed interest in participating in the study received written information, gave schedule opportunities, provided contact details and were assigned to a focus group based on their screening preferences.

### Data collection

**Fig. 2:**
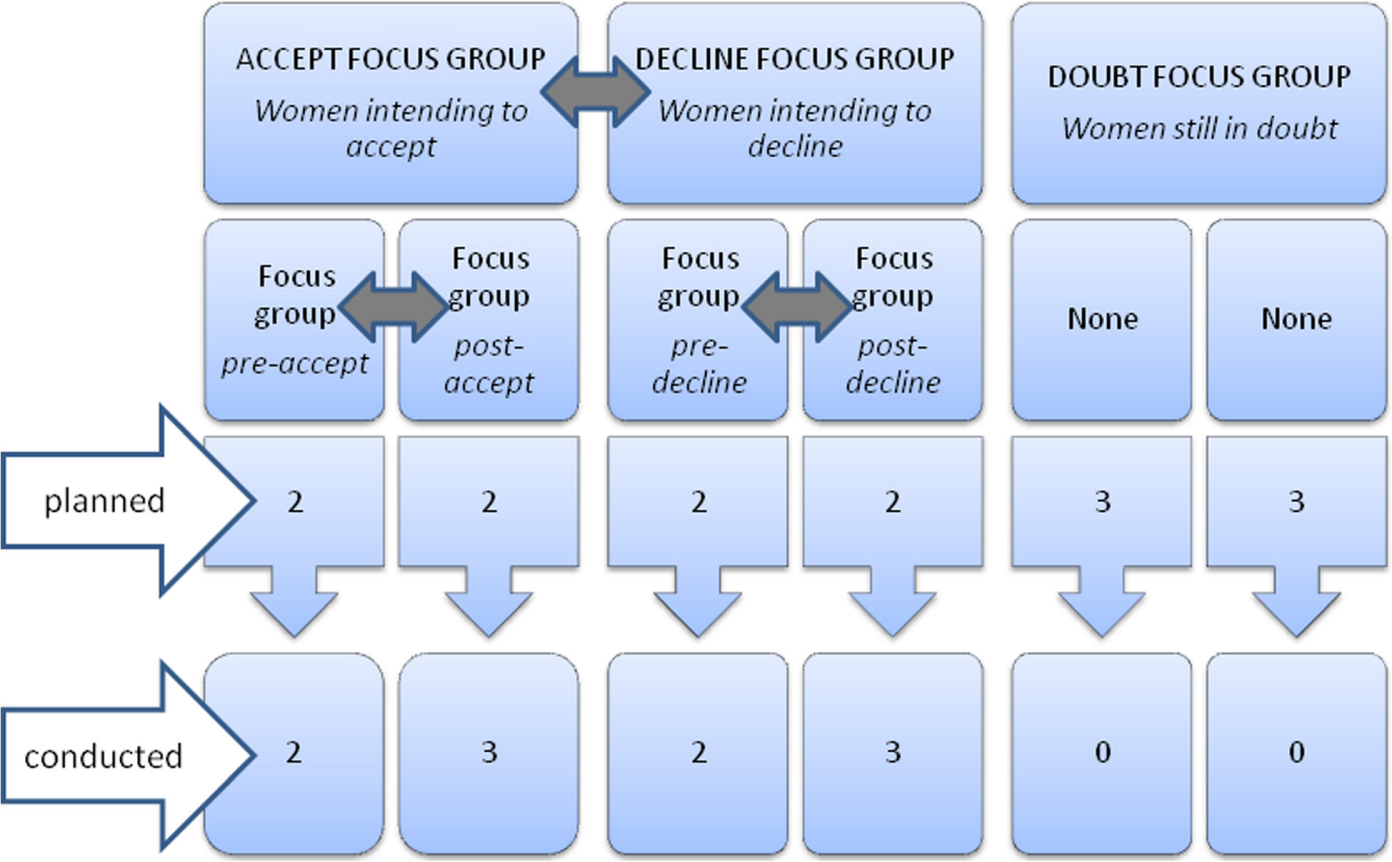
Focus group set up. Intention and conducted. Grey arrows indicate comparison of groups. Focus groups with women intending to accept versus focus groups with women intending to decline. Focus groups with women intending to accept pre-counselling versus focus groups with women intending to accept post-counselling. Focus groups with women intending to decline pre-counselling versus focus groups with women intending to decline post-counselling

As we were interested in which factors impeded or facilitated the use of DSS, women were assigned to specific focus groups stratified according to their intention to utilise DSS (to accept DSS, to decline DSS or in doubt regarding participation). In practice, most women in our study had already made their decision before attending the focus groups, some even had taken the test already (blood test only), but no one had received their test result yet. As discussions could comprise ethical, emotional and social dilemmas, homogeneous groups were set up to create a safe and open environment in which participants would feel free to express their feelings to members of a uniform group [15, 16]. To capture the initial decision making process, four focus groups consisted of women who had not visited a healthcare professional yet (defined as pre counselling groups), of which two focus groups consisted of women who were considering accepting the test (*pre-accept group*), and two focus groups consisted of women who were considering declining the test (*pre-decline group*). For a closer analysis of the impact that professional advice had on the decision-making process, six focus groups were comprised of women who had already visited a healthcare professional (defined as post-counselling groups), of which three groups consisted of women accepting the test (*post-accept group*), and three focus groups consisted of women declining the test (*post-decline group*) (Fig. 2).

We intended to conduct 14 focus groups (Fig. 2), but while recruiting participants we observed that very few women were in doubt and that those who were, already had decided on DSS or otherwise before counselling. Therefore we were unable to conduct any ‘in doubt’ groups. During post-counselling groups, we noticed that the factors mentioned were very diverse and therefore increased the number of ‘accept’ and ‘decline’ groups to three each. In the pre-counselling group we did not experience this explicit diversity and therefore kept to the original plan. In total we performed 10 focus groups with 46 female participants (Fig. 2).

Informed consent was obtained from all participants before the start of the focus group. Before the session, all participants were asked to complete a brief questionnaire to collect information on demographics and a personal history.

All focus groups were performed with a moderator guide. At each session a moderator was present to guide the discussion and a minutes secretary to take notes. The sessions started with the following question: “*Have you thought about participating in prenatal screening for Down syndrome, why or why not?”,* leading to a discussion guided by the moderator. Subsequent questions were used to clarify and probe for more depth guided by the Andersen model. The model was not used to introduce new arguments, but for an in-depth explanation of arguments mentioned. For example, costs were mentioned in all groups, but only superficially. The moderator would then ask if this argument played a role in the decision-making process and how this affected their ultimate decision. During the focus group sessions, the moderator and secretary took notes of implicit observations (such as group interaction, emotions, and atmosphere) and compared and discussed these afterwards. Focus group sessions lasted approximately 180 min, were digitally recorded (audio and video) and transcribed verbatim.

### Analysis

Content analyses were performed using the qualitative software programme Nvivo. Whole group analysis as described by Spencer et al. were used to identify patterns and major themes in the focus group transcripts. The data produced by the groups were used as a whole, and comparisons were made between them (Fig. 2) [17]. Participant-based group analysis was used to deepen the theme of ambivalence, in which the contributions of the participants were analysed separately within the context of the group discussion [17].

**Fig. 3:**
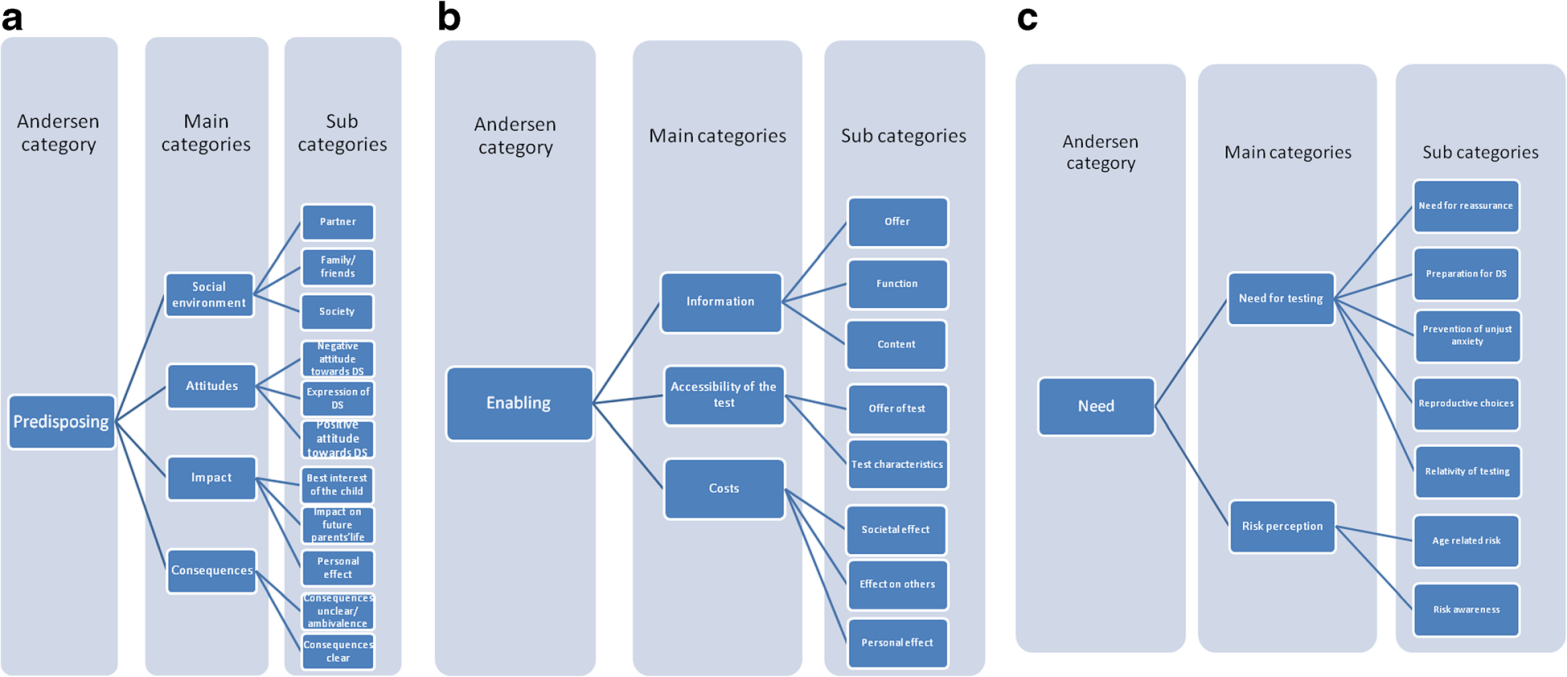
**a** Andersen category predisposing. Most frequently mentioned factors (more than eight times, or in at least four focus groups) were categorised as subcategories, of which main categories were established, identified as Andersen predisposing category. **b** Andersen category enabling. Most frequently mentioned factors (more than eight times, or in at least four focus groups) were categorised as subcategories, of which main categories were established, identified as Andersen enabling category. **c** Andersen category need. Most frequently mentioned factors (more than eight times, or in at least four focus groups) were categorised as subcategories, of which main categories were established, identified as Andersen need category

The focus group transcripts were systematically coded. First, we conducted open coding, in which we assigned initial codes to text fragments. Two researchers with a background in qualitative research (NC and TM) conducted independent analysis of the transcript to reach an understanding on the assigned open codes (subcategories). As a measure of the level of influence, all focus group transcripts were studied at the level of most frequently mentioned subcategories. Subcategories coded more than eight times or mentioned in at least four focus groups (accept or decline focus groups) were studied for further analysis (axial coding). Then, the initial codes were combined by making connections between categories and placing them in a broader context related to the research subject (Fig. 3a, b, c). To improve the quality of the analysis, at various moments interim analyses were discussed among co-authors (JB and HB) with a background in psychology and qualitative analysis. Coding themes, for example, ‘risk awareness’ and ‘age-related risk’ both became subcategories of ‘risk perception’ (Fig. 3c). Finally, the ‘selective coding’ main categories were, when possible, systematically related to categories of the Andersen model of health behaviour (predisposing, need and enabling). For example ‘accessibility of the test’, ‘information’ and ‘costs’ were related to the core category ‘enabling’ (Fig. 3b). Thereafter, all previously analysed transcripts were reviewed to check that their content was consistent with this concept (thematic analysis).

## Results

In general, the focus groups were lively and participants appeared to be open about their arguments and were motivated to participate. All participants were able to formulate arguments and the group interaction contributed to deepen the discussions. The groups intending to accept gave more lively discussions, undoubtedly due to their varied opinions, whereas the decline groups had a higher degree of agreement between participants. Participants were relatively highly educated and the majority had a paid job. The mean maternal age of women who declined was 30 and of those who accepted 32. The majority of participants were Caucasian and were not actively religious. Women declining were more often against abortion (32 %), than those accepting (8 %). Participants’ characteristics are summarised in Table 1.

**Table 1:** Population characteristics

Variable	*Acceptors*			*Decliners*		
	*Pre-counselling*	*Post-counselling*	*Total*	*Pre-counselling*	*Post-counselling*	*Total*
	*n*	*n*	*n*	*n*	*n*	*n*
Total number of women	10	14	24	10	12	22
Maternal age						
*Mean maternal age*	31	33	32	31	29	30
*Maternal age <36*	10	12	22	10	12	22
*Maternal age ≥36*		2	2			
Highest education						
*Low (secondary school)*	1		1			
*Medium (secondary school and vocational education)*	4		4	2	3	5
*High (high vocational education or university)*	5	14	19	8	9	17
Occupation						
*Paid job*	7	12	19	10	11	21
*Unemployed*	2	1	3			
*Disabled*						
*Housewife*	1		1		1	1
*Student*		1	1			
Marital status						
*Married*	6	10	16	7	7	14
*Civil partnership*	3	4	7	3	5	8
*Other (divorced, widowed)*	1		1			
Parity						
*Nulliparous*	5	5	10	3	5	8
*Multiparous*	5	9	14	7	7	14
*Spontaneous abortion*	4	5	9	2	6	8
Religion						
*Active religion*	1		1		2	2
*No active religion*	9	14	23	10	10	20
Experience with Down syndrome						
*Yes*	4	1	5	3	5	8
*No*	6	13	19	7	7	14
Attitude towards abortion						
*In general*						
*Against*	1	1	2	2	5	7
*In favour*	5	12	17	6	5	11
*No opinion*	4	1	5	2	2	4
Personal						
*Yes*		2	2			
*No*	2	1	3	6	3	9
*Depending on situation*	8	11	19	4	9	13
Ethnicity						
*Western*	9	14	23	10	10	20
*Non-Western*	1		1		2	2

### Relative importance of factors of influence

When focus group transcripts were studied at the level of most frequently mentioned factors, the main categories were consequences, attitudes towards Down syndrome, need for testing, risk perception, costs, and accessibility of the test. Subsequently, these categories were compared between women accepting and declining DSS.

### Differences in factors mentioned between women accepting and declining DSS

#### Frequently mentioned arguments by women declining DSS

Women declining DSS did not consider DS a severe enough condition to justify TOP.

“…*we have decided not to participate as we decided to keep the baby, regardless of the test result…” (F502, post-decline group)*

*“So, yes, I would never consider an abortion, as this is my child. And I would try my very best to give it every possible opportunity for a good life” (F404, post-decline group)*

They declined the test as they wanted to prevent potential anxiety arising from possible false positive results.

“*I don’t think I could make decisions on this (Down syndrome), so that’s why I prefer not to be informed, to avoid unnecessary stress” (F404, post-decline group)*

Additionally, as the test result is presented as risk estimation, the test would not provide full reassurance.

*“….I have the impression people look for reassurance which cannot be given, during pregnancy there are so many things that might go wrong (so certainty is impossible)” (F401, post-decline group)*

Costs of testing were, for most women, not a reason to decline, but the additional fee was perceived as extra confirmation that testing was not necessary. There was general agreement that additional costs might result in unequal accessibility.

*“Costs attached to the test do not play a role, as they are not very high. But umm…for me it confirms I do not belong to the high-risk group“(F202, post-decline group)*

Women declining did not believe they were at high risk of carrying a baby with DS; for some, advanced maternal age was not a reason to accept, whereas some young women declined the test because they considered themselves not to be at high risk for DS.

*“I feel young and according to the statistics I am not at high risk… (F904, pre-decline group)”*

#### Frequently mentioned arguments by women accepting DSS

These women wanted to be reassured or to be prepared to care for a child with DS, in the case of diagnosed DS.

*“But if the condition is present, we want to know that in advance” (F305, post-accept group)*

An advanced maternal age was a reason to test, whereas younger women wanted to be reassured with a low-risk test result.

*“I am already 34, people say the older you get, the more chance that complications will arise, I do not want invasive testing due to the risks involved, combined testing is therefore a good option” (F1003, pre-accept group)*

*“There are so many questions… you just want to be reassured…” (F706, pre-accept group, age 26)*

Women accepting the test also weighed up the impact a child with DS could have on their family, but uncertainty about expression and severity of DS made the decision more complicated.

*“…a child with Down syndrome…it depends…there are severe and mild forms of it. To be honest… I haven’t decided what to do in the case of a positive test result…” (F104, post-accept group)*

Costs were not a problem for women accepting, but this factor discouraged others from taking the test. They all agreed that additional costs caused inequality on a social level.

#### Ambivalence

Strikingly, the majority of women accepting the test offer showed uncertainty regarding the consequences of a screen positive result.

*“…there is no harm in taking the test, but I am not sure what I’d do with a positive test result…” (F707, pre-accept group).*

*“…supposing we have a positive test result…we haven’t thought about that yet” (F301, post-accept group)*

TOP in the case of diagnosed DS was not self-evident and for some women diagnostic procedures were unacceptable due to their iatrogenic risk of pregnancy loss.

We were able to identify two groups of women within the accept group: the majority of women were categorised as ‘ambivalent’, since they were of the opinion that TOP in the case of diagnosed DS was not self-evident. A smaller group of women were categorised as ‘evident’, as they were not willing to accept a child with DS and would seriously consider TOP in the case of a diagnosed DS foetus (Table 2).

**Table 2:** Quotes related to factors mentioned by women accepting DSS, categorised as ‘ambivalent’ or ‘evident’

Accept	
*Ambivalent*	
Reassurance/certainty	*“I was looking for certainty, to hear that everything was OK, and I made use of every opportunity to have this certainty, so yes…that also influenced my decision (F301 Post-accept)”*
Preparation	*“But I think, it is good to be prepared when expecting a child with Down syndrome, as far as possible…. (F702 Pre-accept)”*
Guidance	*“she (the midwife) gave me the information leaflet and advised me to read it, as if she expected me to participate… as if she hoped or wanted me to do it… (F104 post-accept)”*
Age	*“For me this (my age) does not play a role, although I find it a reassuring thought that I am still young and am not at risk regarding this (Down syndrome)… (F603 post-accept)”*
*Evident*	
Individual reproductive choices	*“For me, having a choice just feels good (F102 post-accept)”*
	*“We(me and my partner) choose to have kids, given that we want to bring up independent human beings […] now I can make this choice, when a baby is born there is no choice (F101 post-accept)”*
Age	*“… in all these information leaflets you see 36 years of age. And when you start heading towards 40, it really gets dramatic…and I am not far from 40 so… well… yes… (F601 post-accept)”*
	*“well, I am 40, so we are thinking about… yes… what the risks are, and I think the risks increase the older you get (F306 post-accept)”*

### Differences in factors mentioned between pre-counselling and post-counselling groups

To determine the possible influence that the information provided by obstetric caregivers had over the final choices of the participants, we compared pre- and post-counselling groups. Both groups were formed from the groups of women intending to accept and intending to decline (Fig. 2).

#### Pre-counselling versus post-counselling focus groups of women intending to decline

Pre-counselling, women intending to decline had mainly found their information through the information leaflet they received before their first visit, the internet, and an earlier pregnancy. Post-counselling, these women more often referred to information from tables with age-related risks mentioned in the information leaflets. They often recalled perceived support for their decision by their midwife, due to a low a priori risk (Table 3).

**Table 3:** Overview of categories of pre-counselling and post-counselling groups, within accept or decline groups

Andersen categories	Main categories	ACCEPT		DECLINE	
		Pre-counselling	Post-counselling	Pre-counselling	Post-counselling
Predisposing	*Attitude*	*“for me, Down syndrome is the least severe of all possible disabilities, as far as you should call it a disability, a child could have (F702 pre-accept)”*	*“if a child is still young, whether it has Down syndrome or any other child with special needs, it is relatively easy to take care of, but if they get older […] and yes… then there is an adult that is unable to live independently (F104 post-accept)”*		
		*“such a child can provide so much love and can enjoy life in his or her own special way. That is how I see children with Down syndrome, they enjoy life (F1003 pre accept)”*			
			*“if we accept the birth of a child with Down syndrome, it means that our oldest child will get the responsibility after we have died. Now, we have this opportunity to make a choice and prevent my daughter from being a future family care giver (F302 post-accept)”*		
Predisposing	*Consequences*	*“…in the case of a screen positive result, I haven’t decided what to do yet. First, the test, and then we will see what to do next…at least testing doesn’t harm anyone (F707 pre-accept)”*	*“in the case of a screen positive result I will decide to proceed with invasive diagnostics, needless to say…more knowledge through measurement (F603 post-acc)”*		
			*“if you participate in a test you need to be aware of the possible consequences […] yes, we have talked about that extensively. We have decided if the test result is not good we will decide to have a TOP (F101 post-accept)”*		
Enabling	*Information given by counsellor*	*“I received information from a couple that had been pregnant before (F701 pre-accept)*	*“the midwife asked me if I wanted information on DSS. Then I replied that I already had all the information and wanted the test. So that is it… (F602 post-accept)”*		*“And she (the midwife) gave me that bit of extra confirmation by saying “I would not do it, if I were you” If she had said the contrary I would certainly have reconsidered my decision again…yes” (F405 post-decline)”*
		*“first I searched for information on the web, and I also spoke to a friend that recently did the test (F1003 pre-accept)”*			
					*“And then she (the midwife) said, actually she influenced us a bit: “it all looks good and besides the test could also cause a lot of extra stress (F401 post-decline)”*
Need	*Risk perception*		*“the midwife said: ‘you are 35, you could take the combined test’. So we said yes… why not? If it is possible? (F104 post-accept)”*	*“I am 30 years of age, and I am young, so therefore not at increased risk for Down syndrome. And besides I do not need to know everything in advance (F902 pre-decline)”*	*“I filled in the intake-questionnaire, that is when the midwife said: ‘normally I do not see such a healthy list’. I don’t smoke, drink. But I am 30 […] first signs were reassuring, the scan looked good (F401 post-decline)”*
			*“in the Netherlands women are strongly advised to have children at an early age, not only for Down syndrome, but for your fertility as well….yes… it is strongly advised… not sure by whom… but it is, maybe your doctor or the media (F301post-accept)”*		
				*“for me the decisive reason to decline is my young age and I do not have the feeling I am at risk (F904 pre-decline)”*	
					*“… I have studied the statistics…because I was curious to learn about my personal risk (F204 post-decline)”*
				*“one of the influencing factors is age, so yes… I do not expect to have an increased risk […]so that is why testing would not add anything (F803 pre-decline)*	

#### Pre-counselling versus post-counselling focus groups of women intending to accept

Pre-counselling, these women expressed a more positive attitude towards DS, while women post-counselling more often considered diagnostic testing and TOP in the case of a confirmed DS. Information during counselling was considered concise, but despite this conciseness, attention was given to the pros and cons of participation and most women recalled questions such as ‘what to do in the case of a screen positive result’. Women pre-counselling found information on the internet or through family/friends; post-counselling, they mainly recalled information from their midwife or information leaflet. Risk interpretation was only discussed in the post-counselling groups and interpretation of the numbers was considered complicated and confusing. Women in both groups referred to their age-related risk in general, it was only in the post-counselling groups that older women recalled their age-related risk as a factor mentioned by their healthcare provider or that they had read it in the information leaflet. Women perceived this as a recommendation to participate in DSS and it made them a little anxious (Table 3).

#### Pre-counselling versus post-counselling focus groups: counsellor effect

Women declining referred to perceived support in decision-making by their healthcare professional due to their low a priori risk (Table 3). They recalled verbal confirmation of their young age, healthy lifestyle and good ultrasound results, which was interpreted as an affirmation of their reasoning.

“*And when I said no, she (the midwife) said: well…I think since you are so young…your chances of having an affected child are low. In this way she gave me confirmation that I had made the right choice…and I am happy with that, especially if a professional says it* (F402, post-decline)”

Women accepting more often expressed strong arguments with regard to the consequences of the test in the case of a screen positive result. They were asked if they had considered possible consequences of a screen positive result. In this way, obstetric caregivers tried to support them in analysing the pros and cons of participation. Most women thought this was helpful, but some felt they had to defend their decision and did not feel supported.

*“And then she (the midwife) said, but if you decide to participate, you have to think of what…well…if it is not a reassuring test result…what will your decision be then (*F104, post-accept*)”*

## Discussion and conclusion

### Discussion

Our data showed that women in this study incorporate different factors when making their decision. Besides individual considerations, women incorporated screening policy characteristics into their decision. For example, older women experienced being encouraged to use DSS, while younger women being experienced to be discouraged to make use of the screening offer. These findings, together with the finding that a diagnosed DS was not seen as an obvious reason to terminate their pregnancy, possibly account for the low Dutch uptake rates.

Women value their age (predisposing) by what is generally known (external environment) and translate this into their personal age-related risk (perceived need) [7, 13, 18, 19]. As demonstrated in this study, age-related risk played a major role in the decision making process, also reflected in the strong correlation between DSS uptake and age in the Netherlands [20, 21]. This association derives from the connection between advanced maternal age and DS [22, 23]. However, the risk assessment of foetal Down syndrome based on maternal age alone is considerably less accurate than determining an individual risk based on first trimester combined testing [24]. But, as shown in this study, women based the severity of their risk mainly on their age and felt supported by the system in their considerations. For example, by the information on age-related risk in the leaflet and the information received from their healthcare professional.

Specific characteristics of the Dutch screening programme, such as the age-related risk and reimbursement policy, appear to have framed the offer, confirming women in their considerations regarding age-related risk. In particular, being discouraged to participate and the perceived need to defend their decision (enabling) were recalled by young women accepting and might be a reflection of this. In contrast, perceived confirmation of their low risk by screening characteristics (enabling) endorsed the implicit preferred choice towards non-participation for young women.

To understand the context of the current screening programme, some reflection is needed on the period prior to the implementation of the programme. From 1991 until 2004, an extensive public debate took place between government, professional groups, patient organisations and the Health Council (the main advisory organisation to the Ministry of Health in the Netherlands). Some arguments put forward in the discussion against screening were: fear of ‘genetic cleansing’ via the promotion of termination to prevent the birth of disabled children, and fear of an increased medicalisation of pregnancy creating ‘unnecessary anxiety’ in pregnant women [25–28]. Due to of these arguments, the government had to develop a screening policy in which equal access was guaranteed, while preventing pregnant women from routine testing. By offering the service to everyone, equal access was guaranteed but, contrarily, a restraint policy was implemented by integrating ‘the right not to know’ and an additional fee for younger women. The screening programme, as it is now, is unique to Europe [6, 8] and in line with the system in which pregnancy and delivery are considered as normal physiological processes.

Although earlier studies have demonstrated that obstetric caregivers’ attitudes are of no influence on future parents’ choices [10, 29], our data made it clear that they acted consistently with the system’s frame of age-related risk. Young women declining recalled support by their healthcare professional for their decision, whereas young women accepting recalled support in balancing ethics (enabling). Since the majority of Dutch pregnant women start their pregnancy with a midwife, it would be useful to take a closer look at the strategies used to integrate medical screening information in their consultations (see for instance Martin et al. [30]). As described by Rosman [31], obstetric caregivers, in this study midwives, appear to switch from ‘alarming’ biomedical messages to ‘reassuring words’ to manage the anxiety induced by their instructions and to keep control over their low medicalised consultation. Reflecting on a woman’s young age and their a priori low risk for foetal anomalies indicated the use of reassuring words. Remarkably, this only applied to women of a younger age. Perceived recommendation to participate in DSS for older women in this study is in line with the findings of Rosman. In that study it was demonstrated that midwives ‘insisted’ on a woman’s increased risk of having a DS baby and their possibilities of diagnostic testing [31].

The inability to set up ‘in doubt’ groups could indicate that most women decided on utilisation before counselling [29, 32]. But the expression of ambivalent feelings towards subsequent consequences, in the case of a test positive result (e.g. invasive diagnostic testing, TOP) might reflect residual feelings of doubt. For some women, attitudes towards DS might not be seen as relevant at this stage of testing [33], reflected in the “need to be reassured” or “prepared” as important reasons to accept the test offer. Although it is recommended that women make well-informed autonomous choices, feelings of doubt have also been described in earlier studies [34, 35]. Such feelings could undermine the making of informed autonomous choices [35], but there have also been suggestions that ambivalence contributes to weighing pros and cons more thoroughly [36]. Incorporating uncertainties about expression and severity of DS in the decision and considering the impact of a child with DS on their family indicated that, in our study, ambivalence could be seen as a supporting factor in balancing social and emotional dilemmas. The design of the Dutch screening programme is probably supportive in these considerations.

Due to the nature of this type of research, the participants generally had a higher degree of education, often originated from Western countries, spoke Dutch and were on average somewhat older which may limit the findings of our study. Additionally, the number of religious women in this study was low, while research has demonstrated that religious conviction plays an important role in decision-making [13, 20]. To create a safe environment, we organised focus groups with women only and groups had a homogenous set up [15, 16]. Information on the role partners played in the decision-making process was based on the pregnant women’s perceptions, which might be different from their partners’ opinions. Homogenous groups were set up to create a safe environment, which is an advantage of this approach, but might have undermined the discussion on contrasting arguments. The inability to form ‘in doubt’ focus groups may have restricted the discussion on the subject of doubt, but the finding of ambivalence and further investigation gave a more profound impression of the deliberations in the decision-making, characterised by feelings of hesitance.

### Conclusions

In conclusion, different factors are influential in the DSS process. Women incorporate individual factors and are aware of ethical implications. However, for some, choices are partially determined by the system in which they are offered. Women base the severity of their risk on their age and feel that their considerations are sustained by the system.

Future research should further focus on generalisability of these findings. Therefore, the effect of system characteristics on individual decision-making should be tested in large representative groups of women. The conflicting restrictions of the programme seem to encourage young women not to participate, which is inconsistent with the principle of autonomous decision-making. In January 2015 the Dutch reimbursement policy on prenatal screening changed. A fee now has to be paid for the combined test by all pregnant women (regardless of age) as a means to treat all pregnant women alike. The effect on accessibility remains unclear given the restrictive effect due to this new financial threshold for older pregnant women. Policy makers should be aware of these (unintended) side effects, and screening programmes aimed at informed and autonomous decision-making should ideally be offered without conflicting restrictions.

Decision-making in Down syndrome screening should be based on the personal interest in knowing the individual risk of having a child with Down syndrome and participation should not be influenced by system characteristics. To support women in informed and autonomous decision-making, policy makers and obstetric caregivers should therefore: obstetric caregivers 1) inform all women that every person has their own individual risk on having a child with DS, despite their age; and 2) discuss consequences of both accepting and declining testing with all women, regardless of their decision. Only then can obstetric caregivers support women in making the right considerations on accepting or declining DSS. This not only applies to DSS based on classic first trimester biochemical screening in maternal serum, but also to emerging alternative methods such as non-invasive testing based on cell-free foetal DNA in maternal blood. As the consequences of testing could comprise ethical dilemmas, informed and autonomous decision-making is essential and impartial information about the consequences of both accepting and declining testing is of great importance.

## Abbreviations

DS: Down syndrome
DSS: Down syndrome screening
TOP: Termination of pregnancy

## Footnotes

1 Utilisation
